# Aneurysm wall enhancement, hemodynamics, and morphology of intracranial fusiform aneurysms

**DOI:** 10.3389/fnagi.2023.1145542

**Published:** 2023-03-13

**Authors:** Xinyu Liang, Fei Peng, Yunchu Yao, Yuting Yang, Aihua Liu, Duanduan Chen

**Affiliations:** ^1^School of Life Sciences, Beijing Institute of Technology, Beijing, China; ^2^Neurointerventional Center, Beijing Neurosurgical Institute and Beijing Tiantan Hospital, Capital Medical University, Beijing, China; ^3^School of Medical Technology, Beijing Institute of Technology, Beijing, China

**Keywords:** intracranial fusiform aneurysm, hemodynamics, computational fluid dynamics, aneurysm wall enhancement, morphology

## Abstract

**Background and objective:**

Intracranial fusiform aneurysms (IFAs) are considered to have a complex pathophysiology process and poor natural history. The purpose of this study was to investigate the pathophysiological mechanisms of IFAs based on the characteristics of aneurysm wall enhancement (AWE), hemodynamics, and morphology.

**Methods:**

A total of 21 patients with 21 IFAs (seven fusiform types, seven dolichoectatic types, and seven transitional types) were included in this study. Morphological parameters of IFAs were measured from the vascular model, including the maximum diameter (D_max_), maximum length (L_max_), and centerline curvature and torsion of fusiform aneurysms. The three-dimensional (3D) distribution of AWE in IFAs was obtained based on high-resolution magnetic resonance imaging (HR-MRI). Hemodynamic parameters including time-averaged wall shear stress (TAWSS), oscillatory shear index (OSI), gradient oscillatory number (GON), and relative residence time (RRT) were extracted by computational fluid dynamics (CFD) analysis of the vascular model, and the relationship between these parameters and AWE was investigated.

**Results:**

The results showed that D_max_ (*p* = 0.007), L_max_ (*p* = 0.022), enhancement area (*p* = 0.002), and proportion of enhancement area (*p* = 0.006) were significantly different among three IFA types, and the transitional type had the largest D_max_, L_max_, and enhancement area. Compared with the non-enhanced regions of IFAs, the enhanced regions had lower TAWSS but higher OSI, GON, and RRT (*p* < 0.001). Furthermore, Spearman’s correlation analysis showed that AWE was negatively correlated with TAWSS, but positively correlated with OSI, GON, and RRT.

**Conclusion:**

There were significant differences in AWE distributions and morphological features among the three IFA types. Additionally, AWE was positively associated with the aneurysm size, OSI, GON, and RRT, while negatively correlated with TAWSS. However, the underlying pathological mechanism of the three fusiform aneurysm types needs to be further studied.

## 1. Introduction

Intracranial fusiform aneurysms (IFAs) usually have a poor natural history and may lead to ischemia, brainstem compression, and subarachnoid hemorrhage (SAH; Sabotin et al., 2021). Compared with relatively common intracranial saccular aneurysms, IFAs are rare and have different and more complex pathogenesis (Liu et al., 2020). According to the classification proposed by Flemming et al., vertebrobasilar fusiform aneurysms can be divided into three types: fusiform, dolichoectatic, and transitional (Flemming et al., 2005). The potential mechanisms of aneurysm progression and rupture for IFAs remain unclear.

Aneurysm wall enhancement (AWE) on high-resolution magnetic resonance imaging (HR-MRI) has been known as a new potential biomarker of aneurysm instability (Lv et al., 2020; Hashimoto et al., 2021). It has been revealed that AWE is associated with wall inflammation, intraluminal thrombus, and vasa vasorum (Zhong et al., 2021), and that aneurysms with AWE are more likely to grow and rupture (Samaniego et al., 2019). In addition, AWE was also correlated with morphology and hemodynamics, which were also reported to be predictors of aneurysm instability (Lv et al., 2020). As is reported that different AWE patterns may be related to different morphological changes, with focal AWE related to secondary aneurysm formation and circumferential AWE related to whole sac dilation (Hashimoto et al., 2021). Several studies have found a correlation between AWE patterns and hemodynamic characteristics, suggesting the possibility of aneurysm rupture (Lv et al., 2020; Zhang et al., 2021; Zhong et al., 2022). Hemodynamics, which plays an important role in evaluating the risk of aneurysm progression, can be investigated through computational fluid dynamics (CFD) simulations. Certain hemodynamic characteristics, such as low wall shear stress (WSS) and high oscillatory shear index (OSI), are thought to be associated with aneurysm growth and rupture (Sugiyama et al., 2012; Meng et al., 2014; Dabagh et al., 2019). A previous study reported that aneurysms with AWE had lower WSS and average velocity than those without AWE (Khan et al., 2021). Recent studies have further explored the relationship between the location and signal intensity of AWE areas and hemodynamics in saccular aneurysms, as such an association may manifest the interaction between intra-aneurysmal blood flow and aneurysmal wall pathology (Xiao et al., 2018; Hadad et al., 2021; Fu et al., 2022). However, the difference in pathophysiological process between the three IFA types, in combination with the associations between AWE and morphology and hemodynamics in IFAs remains unclear.

In this study, the regions of AWE were extracted and identified on the aneurysm wall to investigate the distribution of AWE on fusiform aneurysms in three-dimensional (3D) space. Computational fluid dynamics (CFD) analysis, demonstrating intra-aneurysmal hemodynamics, was performed on the vascular model containing the fusiform aneurysm. Subsequently, features of AWE, hemodynamics, and morphology differentiated by three IFA types were explored. In the second part, the correlation between the distribution of AWE and morphology and hemodynamic parameters in 3D space was investigated.

## 2. Materials and methods

### 2.1. Patients

Patients with unruptured fusiform aneurysms who underwent HR-MRI at Beijing Tiantan Hospital from May 2017 to November 2021 were prospectively recruited in this study. The exclusion criteria were as follows: (1) patients with contraindications to MRI, (2) image quality was poor and motion artifacts were observed, (3) fusiform aneurysms were not located on the vertebrobasilar artery (VBA), and (4) fusiform aneurysms associated with saccular aneurysms, dissecting aneurysms (Flemming et al., 2005), moyamoya disease, and arteriovenous malformations.

### 2.2. HR-MRI protocol

All of the HR-MRI scans were derived from 3.0 T MR scanners (Trio-Tim, Siemens Healthcare, Erlangen; Discovery 750, GE Healthcare, Milwaukee, WI; Ingenia CX, Philips Healthcare, Best) with a 32-channel head coil. The protocol of HR-MRI images included 3D T1WI (SPACE/CUBE/VISTA) and contrast-enhanced 3D T1WI (SPACE/CUBE/VISTA) with a voxel size of 0.7 × 0.7 × 0.7 mm^3^. After 6 min of the scanning of pre-contrast T1W images, post-contrast T1W images were obtained after the Gd injection (0.1 mmol/kg gadopentetate dimeglumine, Magnevist; Bayer Schering Pharma AG) with the same parameters.

### 2.3. Morphological characteristics

Each vascular model was generated from time-of-flight magnetic resonance angiography (TOF-MRA) in Mimics Research 19.0 (Materialise, Leuven, Belgium) and exported to a stereolithography format for smoothing and trimming in Geomagic Studio 2012 (Geomagic, Research Triangle Park, North Carolina). We extracted the centerlines of vascular models to measure the morphological parameters including maximum diameter (D_max_) and maximum length (L_max_), as we previously reported (Peng et al., 2022a). In addition, the curvature (Zhao et al., 2018) and torsion (Zhao et al., 2018) of aneurysms were also included. D_max_ was defined as the maximum distance between the centerline and the vascular model surface, while L_max_ was defined as the length of the centerline of the fusiform aneurysm part (Peng et al., 2022a). Using our custom scripts in MATLAB R2020b (MathWorks, Natick, Massachusetts, United States), the curvature and torsion of the centerline were calculated, which represent the degree of bending and twisting of the curve, respectively.

### 2.4. AWE analysis

**Figure 1:**
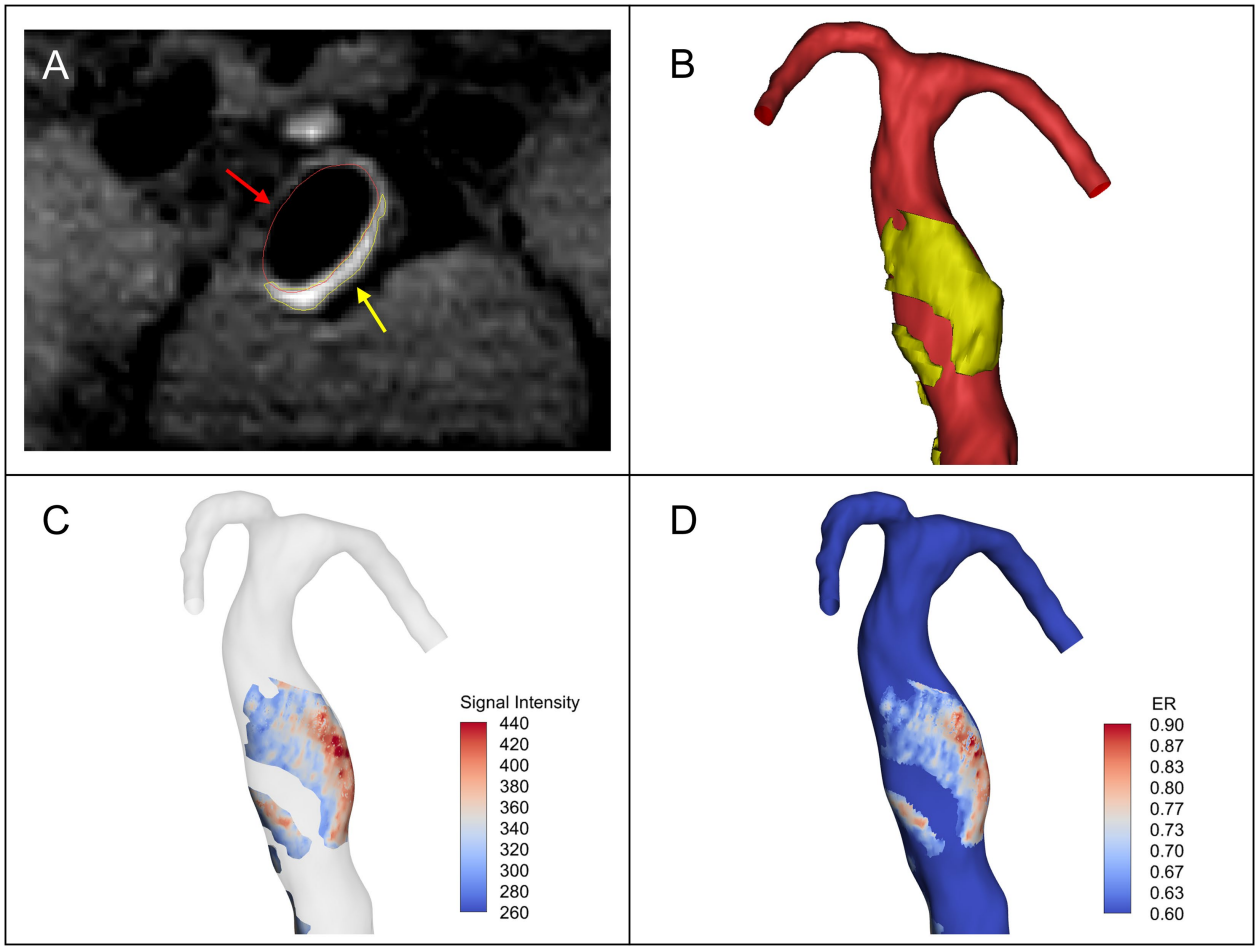
**(A)** The vascular lumen region (red arrow) and the AWE region (yellow arrow) segmented in the registered post-contrast high-resolution magnetic resonance imaging (HR-MRI). **(B)** Vascular model (red) and 3D model of the aneurysm wall enhancement (AWE) region (yellow). **(C)** The signal intensity of AWE was projected onto the vascular model to identify enhanced regions on the surface of the model. **(D)** The enhancement ratio (ER) was calculated by normalizing the signal intensity value of AWE at each point on the model surface with the maximum signal intensity value of pituitary stalk.

Post-contrast HR-MRI was registered to TOF-MRA in 3D-Slicer. Based on the registered HR-MRI images, the maximum signal intensity of the pituitary stalk was calculated. To the best of our knowledge, in some previous studies, an aneurysm with enhancement was defined as having an aneurysm-to-pituitary stalk contrast ratio (CR_stalk_) of 0.6 or greater (Roa et al., 2020; Sabotin et al., 2021). Similarly, in this study, the maximum value of pituitary stalk signal intensity multiplied by 0.6 was used as the lowest threshold to segment the AWE regions in the registered HR-MRI by using Mimics Research 19.0 (Figures 1A,B). When segmented, the AWE segmentation was guaranteed to be on the vessel wall outside the lumen. For each patient, we also calculated the maximum CR_stalk_ of the aneurysm (CR_max_). The AWE segmentation was then projected onto the vascular model to determine enhanced regions on the aneurysm surface, while the signal intensity of AWE was assigned to each point on the enhanced regions (Figure 1C). The remaining regions were defined as non-enhanced regions. The surface area of the enhanced regions (enhancement area) was calculated subsequently, as well as the proportion of the enhanced regions over the entire fusiform aneurysm surface. The enhancement ratio (ER) was used to quantify the degree of enhancement for each point on the aneurysm surface, that is, the signal intensity value of AWE at each point was normalized by the maximum signal intensity value of the pituitary stalk (Figure 1D).

### 2.5. Hemodynamic analysis

All the 3D vascular models were imported into ICEM CFD 2020 R2 (ANSYS, Canonsburg, PA, United States) for meshing. For each model, three layers of wall prism elements were created to obtain precise boundary layer resolution, and the number of generated unstructured mesh elements including triangular, quadrilateral, tetrahedra, and prisms was about 2 million. Mesh independence study was conducted using the model of one case (Supplementary Figure), confirming the rationality of the basic number of mesh elements. Afterward, the created mesh of each model was imported into CFX 2020 R2 (ANSYS, Canonsburg, PA, United States) for CFD simulation by solving the Navier–Stokes equations. The vessel was considered a rigid and non-slip wall. The blood flow was assumed to be a laminar, incompressible Newtonian fluid with a density of 1,066 kg/m^3^ and a viscosity of 0.0035 Pa·s. Pulsatile flow velocity boundary conditions were set at the vascular inlets and pressure boundary conditions at the outlets. The inlet and outlet boundary conditions based on cardiac cycle variation were extracted from @neufuse software, where the database of human blood vessel boundary function information was contained. Depending on the inlet and outlet locations, the corresponding boundary conditions were used. For the different models investigated, the inlet peak Reynolds number was 500–700. The time-step size was set to 0.008 s. To ensure numerical stability, three cardiac cycles with 300 time-steps were simulated, and then the results from the last cardiac cycle were selected to calculate the hemodynamic parameters of the fusiform aneurysm.

During the simulation, we computed several hemodynamic parameters including time-averaged wall shear stress (TAWSS; Diagbouga et al., 2018), oscillatory shear index (OSI; Longo et al., 2017), gradient oscillatory number (GON; Shimogonya et al., 2009), and relative residence time (RRT; Xiang et al., 2011). TAWSS indicates the average magnitude of tangential frictional stress exerted by the blood flow on the vessel wall during the cardiac cycle. In addition, the aneurysm TAWSS was normalized by the parent vessel TAWSS to compare TAWSS magnitude among different patients. OSI is a nondimensional parameter that measures the degree of directional variation of the WSS vector during the cardiac cycle. GON quantifies the directional oscillation of the spatial wall shear stress gradient and can reflect the blood flow disturbance. RRT reflects the residence time of blood near the vessel wall. These parameters are defined as:

$$TAWSS=\frac{1}{T}\overset{T}{\underset{0}{\int }}\left|WSS_{i}\right|dt$$

$$\begin{array}{c} OSI=\frac{1}{2}\left\{1-\frac{\left|\int_{0}^{T}WSS_{i}dt\right|}{\int_{0}^{T}\left|WSS_{i}\right|dt}\right\} \end{array}$$

$$\begin{array}{c} GON=1-\frac{\left|\int_{0}^{T}Gdt\right|}{\int_{0}^{T}\left|G\right|dt} \end{array}$$

$$\begin{array}{c} RRT=\frac{1}{\left(1-2\times OSI\right)\times TAWSS}=\frac{1}{\frac{1}{T}\left|\int_{0}^{T}WSS_{i}dt\right|} \end{array}$$

where $WSS_{i}$ is the instantaneous WSS vector, T is the duration of one cardiac cycle, G is the spatial wall shear stress gradient vector.

### 2.6. Statistics

All statistical analyses were performed using SPSS Statistics 23 (IBM, Armonk, New York, United States). Variables were tested for normality, and variables satisfying a normal distribution were expressed as mean ± SD. ANOVA was used to compare the AWE, morphological and hemodynamic characteristics of the three types of fusiform aneurysms. The Mann–Whitney U test was used to analyze the differences in hemodynamic parameters between enhanced and non-enhanced regions. The correlation between AWE and hemodynamic parameters on the aneurysm surface was investigated by Spearman’s correlation analysis. *p* value <0.05 was set as the criterion for statistical significance.

## 3. Results

### 3.1. Characteristics of three different IFA types

From the prospective database of Beijing Tiantan Hospital, we identified and analyzed 21 patients with 21 fusiform aneurysms located on the VBA. These fusiform aneurysms were classified into three types, with seven cases in each type. We analyzed the differences in morphological parameters, AWE characteristics, and hemodynamic variables among three IFA types (Table 1). The results showed that D_max_ (*p* = 0.007) and L_max_ (*p* = 0.022) were related to different types. The transitional type had the largest D_max_ of 13.44 ± 3.10 mm, followed by the dolichoectatic type (9.92 ± 2.13 mm) and the fusiform type (8.66 ± 2.32 mm). The transitional type also had the largest L_max_ (31.72 ± 16.73 mm), and the fusiform type remained the smallest (14.16 ± 4.07 mm).

**Table 1:** Characteristics of three different IFA types.

Variables	Fusiform (*n* = 7)	Dolichoectatic (*n* = 7)	Transitional (*n* = 7)	*p* value
D_max_ (mm)	8.66 ± 2.32	9.92 ± 2.13	13.44 ± 3.10	**0.007** ^*^
L_max_ (mm)	14.16 ± 4.07	23.61 ± 6.65	31.72 ± 16.73	**0.022** ^*^
Max curvature (mm^−1^)	0.12 ± 0.03	0.11 ± 0.06	0.15 ± 0.09	0.632
Mean curvature (mm^−1^)	0.07 ± 0.03	0.06 ± 0.01	0.07 ± 0.02	0.229
Max torsion (mm^−1^)	0.58 ± 0.21	0.55 ± 0.22	0.66 ± 0.25	0.662
Mean torsion (mm^−1^)	0.23 ± 0.08	0.23 ± 0.12	0.19 ± 0.08	0.607
CR_max-IA_	1.11 ± 0.34	1.14 ± 0.15	1.04 ± 0.12	0.705
Enhancement area (mm^2^)	51.72 ± 42.91	207.40 ± 98.06	226.00 ± 102.34	**0.002** ^*^
Proportion of enhancement area	0.17 ± 0.11	0.40 ± 0.14	0.28 ± 0.11	**0.006** ^*^
TAWSS_max_ (Pa)	1.13 ± 0.86	1.42 ± 1.08	1.52 ± 1.00	0.742
TAWSS_mean_ (Pa)	0.33 ± 0.22	0.28 ± 0.18	0.20 ± 0.10	0.386
OSI_max_	0.47 ± 0.01	0.48 ± 0.01	0.48 ± 0.01	0.101
OSI_mean_	0.10 ± 0.03	0.12 ± 0.05	0.12 ± 0.03	0.602
GON_max_	0.89 ± 0.04	0.91 ± 0.03	0.91 ± 0.03	0.641
GON_mean_	0.20 ± 0.07	0.19 ± 0.07	0.23 ± 0.04	0.552
RRT_max_	97.45 ± 38.82	202.70 ± 179.05	256.00 ± 208.10	0.197
RRT_mean_	4.46 ± 2.40	7.01 ± 6.57	6.21 ± 3.07	0.553

* Significant differences (*p* < 0.05). TAWSS, time-averaged wall shear stress; OSI, oscillatory shear index; GON, gradient oscillatory number; and RRT, relative residence time. The bold values provided in table mean the *p*-value is less than 0.05, meaning the statistical results show significant differences.

There was no significant difference in CR_max_ (*p* = 0.705) among the three types. However, enhancement area (*p* = 0.002) and proportion of enhancement area (*p* = 0.006) were significantly different. The transitional type had the largest enhancement area (226.00 ± 102.34 mm^2^), while the dolichoectatic type had the largest proportion of enhancement area (0.40 ± 0.14). Enhancement area and the proportion of enhancement area were the smallest in the fusiform type.

The maximum and mean values of hemodynamic variables including TAWSS, OSI, GON, and RRT were also compared among the three types, but no significant differences were found (Table 1).

### 3.2. Correlation of AWE with hemodynamic characteristics and morphological parameters

**Figure 2:**
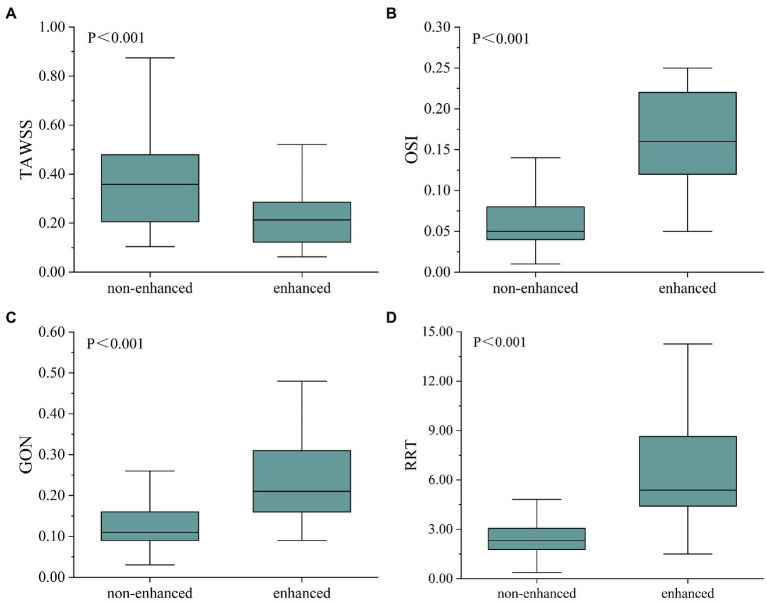
Box diagram showed the comparison of hemodynamic parameters between non-enhanced and enhanced regions. TAWSS was higher in the non-enhanced region **(A)**, whereas OSI **(B)**, GON **(C)**, and RRT **(D)** were higher in the enhanced region. TAWSS, time-averaged wall shear stress; OSI, oscillatory shear index; GON, gradient oscillatory number; and RRT, relative residence time.

**Figure 3:**
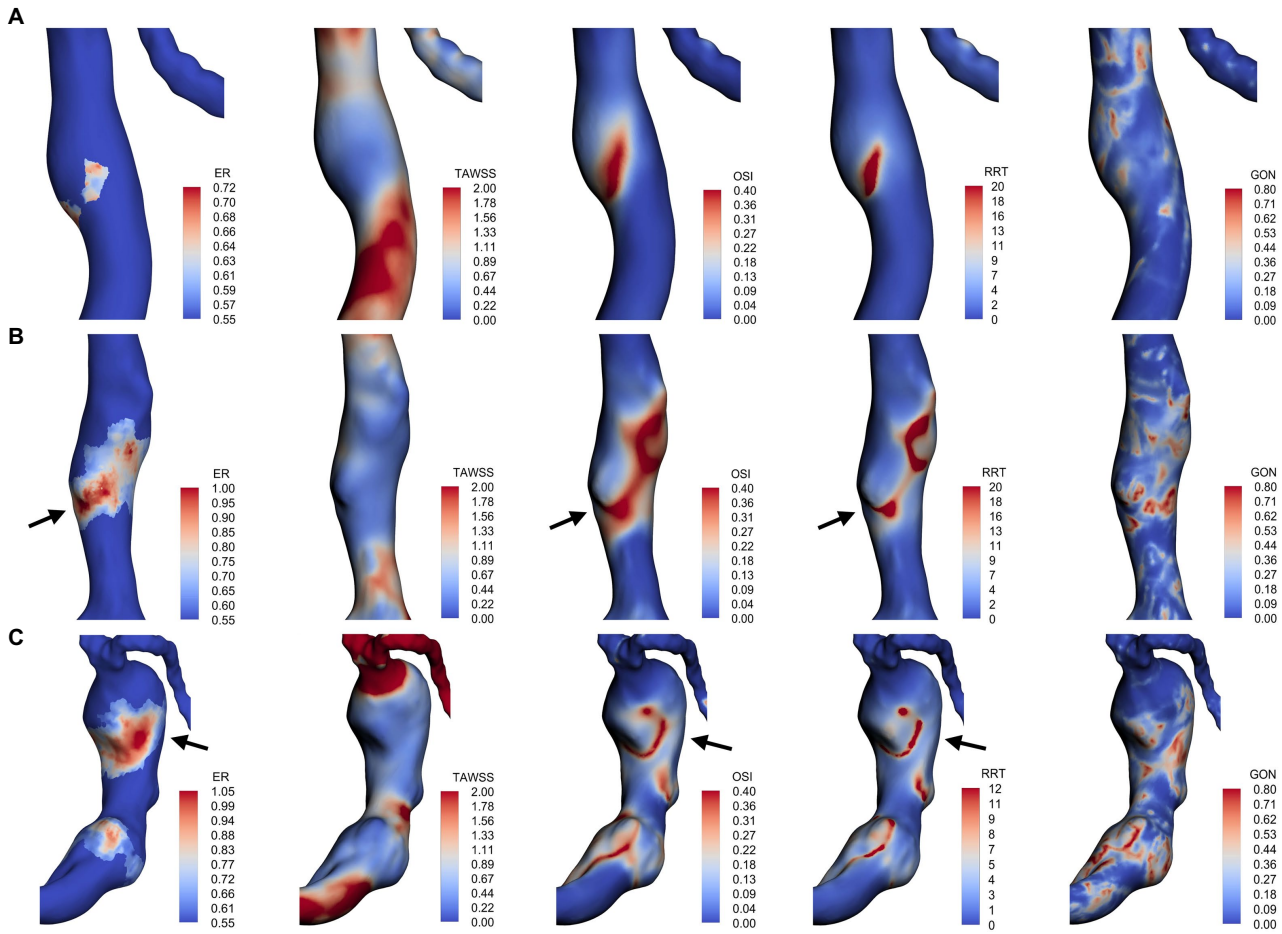
AWE distribution and hemodynamic characteristics of fusiform type **(A)**, dolichoectatic type **(B)**, and transitional type **(C)**. AWE was found in areas with low TAWSS in all three types. Some locations with higher AWE showed higher OSI and RRT (black arrows).

For each fusiform aneurysm, we further investigated the relationship between AWE (enhanced/non-enhanced) and hemodynamic characteristics in specific regions of the aneurysm. The mean values of hemodynamic variables were used to study the enhanced and non-enhanced regions in each case. It was found that TAWSS was significantly lower, while OSI, GON, and RRT were significantly higher in the enhanced regions compared to the non-enhanced regions (Figure 2). Figure 3 shows the distribution of AWE and hemodynamics in the cases of the three fusiform aneurysm types.

Furthermore, to investigate the correlation between AWE and hemodynamics in 3D space, the hemodynamic parameters and ER values of all nodes on the surface of each aneurysm were extracted and used to analyze the correlation between ER and the numerical magnitude of the hemodynamic variables for each case (Table 2). Overall, ER was negatively correlated with TAWSS, with 14 cases (66.7%) reaching a moderate degree of correlation (*r* > 0.3, *p* < 0.001). One case showed a positive correlation between TAWSS and ER, while Spearman’s correlation coefficient was less than 0.1. In addition, the results revealed that ER was positively correlated with OSI, GON, and RRT. ER was moderately positively correlated with OSI in 17 cases (81%), GON in seven cases (33.3%), and RRT in 16 cases (76.2%).

**Table 2:** Results of Spearman’s correlation analysis between the enhancement ratio (ER) and hemodynamic parameters (*p* < 0.001).

IFA type	Case	TAWSS	OSI	GON	RRT
Fusiform	01	−0.257	0.315	0.208	0.278
	02	−0.738	0.484	0.136	0.701
	03	−0.635	0.591	0.437	0.636
	04	−0.228	0.131	0.132	0.202
	05	−0.494	0.523	0.355	0.524
	06	−0.464	0.395	0.038	0.447
	07	−0.418	0.371	0.217	0.413
Dolichoectatic	08	−0.228	0.202	0.054	0.196
	09	0.051	0.130	0.203	0.034
	10	−0.396	0.442	0.340	0.434
	11	−0.125	0.187	0.178	0.152
	12	−0.556	0.473	0.272	0.530
	13	−0.317	0.307	0.322	0.330
	14	−0.466	0.517	0.259	0.510
Transitional	15	−0.339	0.313	0.372	0.355
	16	−0.378	0.422	0.238	0.413
	17	−0.255	0.442	0.262	0.338
	18	−0.295	0.334	0.080	0.324
	19	−0.492	0.467	0.431	0.498
	20	−0.541	0.482	0.336	0.539
	21	−0.380	0.424	0.199	0.425

TAWSS, time-averaged wall shear stress; OSI, oscillatory shear index; GON, gradient oscillatory number; and RRT, relative residence time.

**Figure 4:**
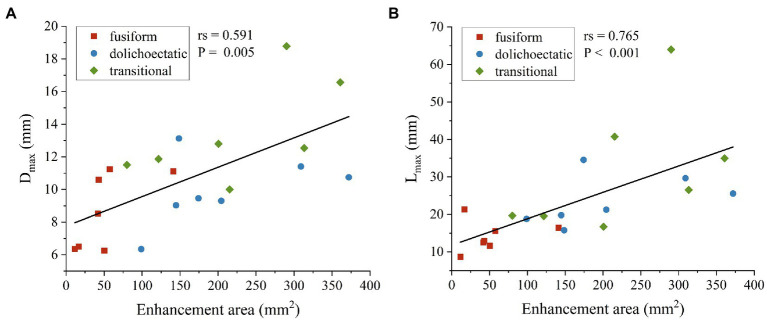
Scatter plots and spearman’s correlation analysis showed that enhancement area was positively correlated with D_max_ **(A)** and L_max_ **(B)** of intracranial fusiform aneurysms.

Spearman’s correlation analysis was also performed between AWE and morphological features. Among all the morphological parameters, only the correlations between D_max_, L_max_, and enhancement area were statistically significant. As shown in Figure 4, enhancement area was strongly positively correlated with D_max_ (rs = 0.591, *p* = 0.005) and L_max_ (rs = 0.765, *p* < 0.001).

## 4. Discussion

As a rare type of intracranial aneurysm, IFA has a complicated pathophysiological process. In this study, based on 3D models, AWE spatial distribution, hemodynamics, and morphology of IFAs were obtained. We attempted to distinguish the different characteristics between the three IFA types. Furthermore, we also sought to understand the potential pathophysiological mechanisms of fusiform aneurysms through the association of spatial AWE and morphology and hemodynamic characteristics. We found the transitional type may have a high risk of instability. We also found that enhancement area was positively correlated with D_max_ and L_max_, respectively. Besides, AWE was negatively correlated with TAWSS and positively correlated with OSI, RRT, and GON.

Different types of fusiform aneurysms seem to have different natural histories (Nasr et al., 2018), so risk factors differentiated by IFA types need to be investigated. Nasr et al. (2016) found that the maximum aneurysm diameter, thrombus, and daughter sac were associated with different types of IFA. We previously reported that AWE was associated with IFA types through a 2D perspective although did not reach statistical significance (Peng et al., 2022a). AWE is considered as a new biomarker of aneurysm instability as it correlated well with atherosclerosis, intraluminal thrombus, neovascularization, and inflammatory cell infiltration (Samaniego et al., 2019). Thus, obtaining the characteristics of overall AWE in 3D space may contribute to understanding the physiological mechanism of aneurysms. In this study, we found the transitional type had the largest enhancement area and the highest D_max_ and L_max_ (Peng et al., 2022a). Compared with fusiform and dolichoectatic types, the transitional type has more complex morphological features, usually accompanied by superimposed expansion (Flemming et al., 2005), which may be related to multi-regional and large-scale AWE. However, it should be noted that although the transitional type had the largest enhancement area in this study, the dolichoectatic type had the largest proportion of enhancement area. This may be due to the fact that the dolichoectatic type is mainly manifested as uniform dilated (Nasr et al., 2018), with a smaller aneurysmal surface area and less local irregular expansion than the transitional type. Cao et al. (2020) found that D_max_ was independently associated with instability of vertebrobasilar non-saccular aneurysms, and we previously found that L_max_ was associated with symptomatic fusiform aneurysms (Peng et al., 2022b). In a follow-up study, the transitional type was found to be more prone to growth and rupture than the other two types (Nasr et al., 2016). Thus, the transitional type may be more vulnerable to poor progression.

Sabotin et al. (2021) explored the pathophysiological mechanism of fusiform aneurysm formation and growth and suggested that the three types of IFA correspond to different CFD patterns. However, after extracting the hemodynamic parameters of the three types by CFD analysis, we found that there were no significant differences in the hemodynamic characteristics of the three IFA types in our study.

It is reported that the aneurysm size was strongly correlated with AWE (Zhong et al., 2020). Hashimoto et al. (2021) conducted a follow-up study of saccular aneurysms with different AWE patterns and found that focal enhancement may lead to secondary aneurysm formation, whereas circumferential enhancement may lead to dilation of the entire aneurysm sac. In our study, we found that enhancement area was strongly positively correlated with D_max_ and L_max_. So fusiform aneurysms with higher D_max_ and L_max_ may generally have a larger area of AWE, while the inflammation and neovascularization processes demonstrated as AWE in turn continue to make the vessel wall more fragile, leading to further enlargement or lengthening of the aneurysm.

Hemodynamics plays an important role in assessing the risk of aneurysm progression. WSS is an essential hemodynamic parameter to study the mechanical effect of blood flow on the vessel wall, which is related to vessel wall remodeling (Boussel et al., 2008; Chalouhi et al., 2013; Cebral et al., 2019). Low WSS and slow blood flow contribute to the infiltration of inflammatory cells (Dabagh et al., 2019), and are prone to endothelial dysfunction (Serrone et al., 2014), which ultimately leads to the degeneration and thinning of the aneurysm wall, as well as the formation of adverse components such as wall thrombosis. OSI reflects the fluctuations and oscillations of WSS. A higher OSI indicates that the direction of the WSS is more prone to change and the vessel wall is subjected to greater oscillatory action. It has been found that low WSS and high OSI may be associated with the growth and rupture of large, atherosclerotic aneurysms (Sugiyama et al., 2012; Meng et al., 2014; Dabagh et al., 2019). In recent years, the association between hemodynamics and AWE has attracted much attention. Khan et al. (2021) studied aneurysms with and without AWE, and the AWE group had lower WSS compared to the non-AWE group. In our study, we found that TAWSS was lower in enhanced regions than in non-enhanced regions, which is consistent with previous studies (Xiao et al., 2018; Larsen et al., 2020; Hadad et al., 2021). Spearman’s correlation analysis showed that AWE was negatively correlated with WSS in most of the cases. As mentioned earlier, low WSS may promote inflammatory cell infiltration, leading to pathological changes in the aneurysm wall. At the same time, increased vascular permeability may facilitate the penetration of contrast media into the aneurysm wall (Zhong et al., 2022). Therefore, the enhanced regions showed the characteristics of low WSS. The combination of hemodynamic parameters and AWE may indicate local lesions on fusiform aneurysms, but the correlation between hemodynamic features and pathological progression of the three IFA types may need further investigation.

The relationship between AWE and other hemodynamic parameters such as RRT, GON, and OSI is still controversial. It has been reported that circumferential wall enhancement is associated with long RRT (Lv et al., 2020; Zhong et al., 2022). RRT, as an index of low or oscillatory WSS, reflects the residence time of blood near the vessel wall. Long RRT may imply low or even stagnant blood flow near the vessel wall, which may favor inflammatory cell infiltration and contrast agent penetration. Regarding GON, high GON is thought to be closely related to aneurysm growth (Shimogonya et al., 2009). Hadad et al. (2021) found that GON was higher in enhanced areas than in non-enhanced areas, but there was no statistical significance. In addition, Fu et al. (2022) found that high-enhanced regions had higher OSI and that OSI was positively correlated with AWE intensity, which is in line with our results. However, Xiao et al. (2018) found that the enhanced regions had lower OSI than the non-enhanced regions. In our study, OSI, RRT, and GON were significantly higher in the enhanced regions than in the non-enhanced regions. Meanwhile, Spearman’s correlation analysis showed that AWE was positively correlated with OSI, RRT, and GON, although the degree of correlation between AWE and GON was weak. Low WSS, high OSI, and long RRT have been associated with thick aneurysm wall regions, such as atherosclerosis and hyperplasia, in previous studies (Furukawa et al., 2018; Cebral et al., 2019). Additionally, pathological and imaging studies have indicated that aneurysm wall thickening accompanied by atherosclerosis is associated with AWE (Shimonaga et al., 2018; Hashimoto et al., 2019; Zhong et al., 2021). This is a possible explanation for the higher OSI and longer RRT in the enhanced region than in the non-enhanced region.

This study has certain limitations. First, HR-MRI images were derived from three different 3 T MRI machines (Simens, GE, and Philip). Whereas, the intrinsic parameters were adjusted to maintain consistency. Second, in our CFD analysis, the vessel wall was assumed to be a rigid wall, and the blood flow was assumed to be a laminar, incompressible non-Newtonian fluid, which was a certain deviation from the real blood vessels *in vivo* and the actual blood flow state. At the same time, the pulsatile flow conditions of healthy subjects rather than specific patients were used, which may have some influence on the hemodynamic results. Finally, the sample size of this study was relatively small and follow-up data were lacking. Therefore, more abundant sample data are needed to study the association between AWE, hemodynamic characteristics, and the growth and rupture of IFAs in the future.

## 5. Conclusion

There were significant differences in AWE distributions and morphological features among the three IFA types, and AWE was associated with the aneurysm size of IFAs. Additionally, AWE was negatively correlated with TAWSS but positively correlated with OSI, GON, and RRT. However, the correlation between hemodynamic characteristics and the underlying pathological mechanism of the three fusiform aneurysm types needs to be further studied.

## Data availability statement

The raw data supporting the conclusions of this article will be made available by the authors, without undue reservation.

## Ethics statement

The studies involving human participants were reviewed and approved by Institutional Review Board of Beijing Tiantan Hospital. The patients/participants provided their written informed consent to participate in this study.

## Author contributions

FP and YunY contributed to conception and design of the study. XL and YutY collected the patient data and analyzed the data. XL and FP wrote the manuscript. DC and AL made the critical revision of the article. All authors contributed to the article and approved the submitted version.

## Funding

This work was supported by Beijing Natural Science Foundation (Z190014) and National Natural Science Foundation of China (82172021 and 62271061).

## Conflict of interest

The authors declare that the research was conducted in the absence of any commercial or financial relationships that could be construed as a potential conflict of interest.

## Publisher’s note

All claims expressed in this article are solely those of the authors and do not necessarily represent those of their affiliated organizations, or those of the publisher, the editors and the reviewers. Any product that may be evaluated in this article, or claim that may be made by its manufacturer, is not guaranteed or endorsed by the publisher.
