# Supplementary material for: Aneurysm wall enhancement, hemodynamics, and morphology of intracranial fusiform aneurysms
Source: Front Aging Neurosci. 2023 Mar 13;15:1145542. doi: 10.3389/fnagi.2023.1145542 (PMC10040612; doi:10.3389/fnagi.2023.1145542)
Supplement: Supplementary file 1 [file Data_Sheet_1.PDF]

*Supplementary Material*

# Aneurysm wall enhancement, hemodynamics, and morphology of intracranial fusiform aneurysms

**Xinyu Liang[1†], Fei Peng[2†], Yunchu Yao[1], Yuting Yang[1], Aihua Liu[2*], Duanduan Chen[1,3*]**

*** Correspondence:** Duanduan Chen: duanduan@bit.edu.cn; Aihua Liu: liuaihuadoctor@163.com

## 1 Supplementary Figures

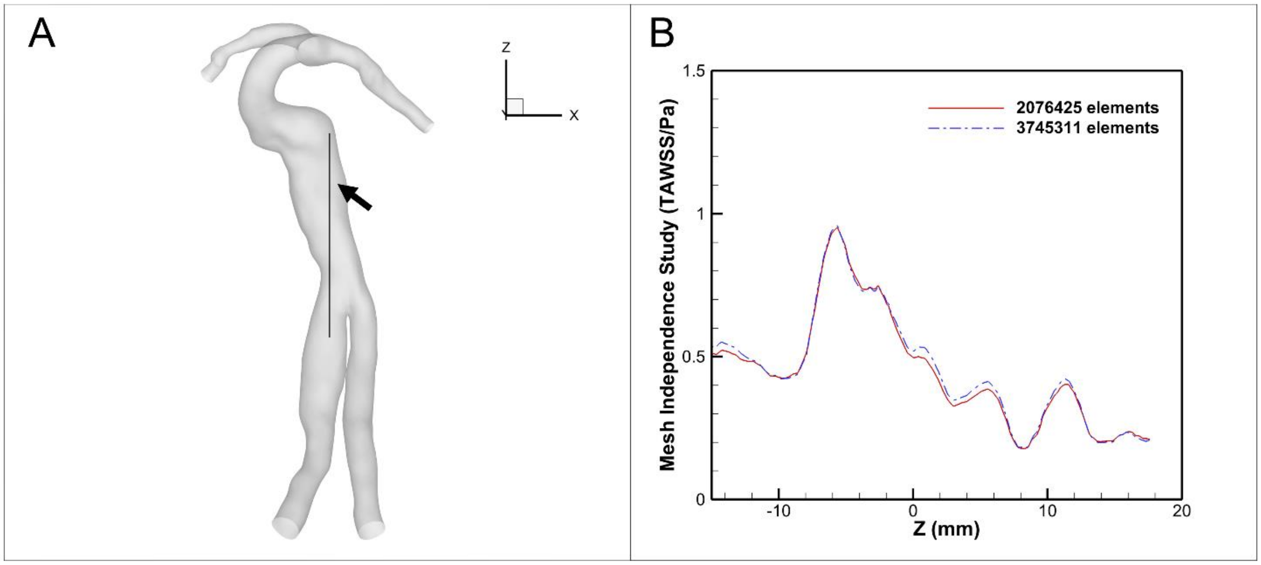


**Supplementary Figure.** Mesh independence study of an example model: (A) A straight line was selected on the model surface to extract the time-averaged wall shear stress (TAWSS) for the mesh independence study. (B) The TAWSS distributed along the straight line was presented on the base mesh (2076425 elements) and the finer mesh (3745311 elements). The maximum discrepancy of the TAWSS between the two meshes is 3.73%. Thus, for the purposes of our study, the base mesh with about 2 million elements is adequate.
